# Optimal timing for lithium levels

**DOI:** 10.12688/f1000research.122507.2

**Published:** 2024-09-16

**Authors:** Kevin J Black

**Affiliations:** 1 Departments of Psychiatry, Neurology, Radiology, and Neuroscience, Washington University in St. Louis School of Medicine, St. Louis, MO, 63110, USA

**Keywords:** lithium, pharmacology, administration & dosage, pharmacokinetics, blood level, concentration

## Abstract

Reddy and Reddy (2014) discuss the optimal timing for lithium levels in patients taking once-daily extended-release lithium formulations. They argue for blood sampling 24 h after the previous dose rather than the standard 12 h. I interpret the data quite differently. The authors start with the assumption that the clinician wants a trough level. I disagree. What one wants is to be able to compare a patient’s lithium level to the large body of published knowledge about lithium dosing. Almost all of that data comes from standard 12-h blood draws with plain (immediate-release) lithium carbonate or lithium citrate. So, the real question of interest is, with extended-release lithium formulations, at what time point does one draw the lithium level to compare most accurately with a standard 12-h blood draw with plain lithium carbonate?

The answer is not obvious because extended-release formulations affect only the absorption and not the excretion of lithium. Their primary benefit is reducing the transient peak lithium serum concentration, not delaying the (already relatively slow) elimination of lithium.

Emami and colleagues (2004) provide the needed data. First they show that 90% of the administered dose of a commercial extended-release formulation (Eskalith CR ^®^) is absorbed by 4 h after a dose, and ~100% is absorbed by 8 h (their Figure 2A). Second, they show that at 12 h after a dose, the blood levels for immediate and extended release formulations are essentially identical (their Figure 3).  Thus 12 h after the previous dose is the ideal time for drawing blood levels for extended-release lithium tablets.

Reddy and Reddy (2014) discuss the optimal timing for lithium levels in patients taking once-daily extended-release lithium formulations. They argue for blood sampling 24 h after the previous dose rather than the standard 12 h. Trainees searching for information on timing of lithium blood sampling have been influenced by that paper’s conclusions. Unfortunately, the journal the original article appeared in does not accept late correspondence, the NLM sadly ended their PubMed Commons experiment, and few physicians use PubPeer. This short note is intended to provide on the 2014 article’s PubMed page a “Comment” link to a more traditional viewpoint.

Reddy and Reddy start with the assumption that the clinician wants a trough level. Rather, what one wants is to be able to compare a patient’s lithium level to the large body of published knowledge about lithium dosing. Almost all of that data comes from standard 12-h blood draws with plain (immediate-release) lithium carbonate or lithium citrate. So, the real question of interest is, with extended-release lithium formulations, at what time point does one draw the lithium level to compare most accurately with a standard 12-h blood draw with plain lithium carbonate?

The answer is not obvious because extended-release formulations affect only the absorption and not the excretion of lithium. Their primary benefit is reducing the transient peak lithium serum concentration, not delaying the (already relatively slow) elimination of lithium.

Emami et al. (2004) provide the needed data. First they show that 90% of the administered dose of a commercial extended-release formulation (Eskalith CR ^®^) is absorbed by 4 h after a dose, and ~100% is absorbed by 8 h (their Figure 2A). Second, they show that at 12 h after a dose, the blood levels for immediate and extended release formulations are essentially identical (their Figure 3). Thus 12 h after the previous dose is the ideal time for drawing blood levels for extended-release lithium tablets.

## Data availability

There are no data associated with this article.
