# Supplementary material for: Optimal timing for lithium levels
Source: F1000Res. 2024 Sep 16;11:779. Originally published 2022 Jul 12. [Version 2] doi: 10.12688/f1000research.122507.2 (PMC11773131; doi:10.12688/f1000research.122507.2)
Supplement: Peer review history

# Reviewer report: Reviewer response for version 2

**DOI:** 10.5256/f1000research.171732.r356872

**Published:** 2025-01-27

**Authors:** Seyyed Nassir Ghaemi

**Affiliations:** 1 Harvard Medical School, Tufts University, Boston, USA

I fully agree with Dr Black's analysis and description. The prior article by Reddy and Reddy was mistaken and misleading. No revision is needed of this correct critique. 

 The half life of lithium does not change whether it is immediate release or extended release. In both cases it is about 24 hours. Yet the level is done at 12 hours based on 75 years of research using that timing to correlate with clinical toxicity.

Are arguments sufficiently supported by evidence from the published literature or by new data and results?

Yes

Is the conclusion balanced and justified on the basis of the presented arguments?

Yes

Is the rationale for commenting on the previous publication clearly described?

Yes

Are any opinions stated well-argued, clear and cogent?

Yes

Reviewer Expertise:

Bipolar illness, psychopharmacology

I confirm that I have read this submission and believe that I have an appropriate level of expertise to confirm that it is of an acceptable scientific standard.

# Reviewer report: Reviewer response for version 1

**DOI:** 10.5256/f1000research.134501.r286092

**Published:** 2024-06-28

**Authors:** Dean MacKinnon

**Affiliations:** 1 Johns Hopkins University School of Medicine, Baltimore, MD, USA

This is a brief correspondence to address somewhat dated reports that suggest altering practice with regard to the timing of therapeutic lithium levels. The author addresses a pharmacologic conjecture that a 24 hour level rather than the standard 12 hour level might be preferable, countering that conjecture with a sensible argument based on clinical utility and data to back it up. A previous commenter had reservations because the critique seemed untimely and thought there could be more data to back it up. While these points are true, I do not see it as my role to question the timing of indexing, and I think the data are sufficient to confirm a point that is consistent with standard clinical practice already.

Are arguments sufficiently supported by evidence from the published literature or by new data and results?

Yes

Is the conclusion balanced and justified on the basis of the presented arguments?

Yes

Is the rationale for commenting on the previous publication clearly described?

Yes

Are any opinions stated well-argued, clear and cogent?

Yes

Reviewer Expertise:

Mood disorders, psychiatric genetics, education, history

I confirm that I have read this submission and believe that I have an appropriate level of expertise to confirm that it is of an acceptable scientific standard.

# Response

**Published:** 2024-06-28

**Authors:** Kevin J Black

**Affiliations:** Washington University in St Louis, St. Louis, Missouri, USA

Thank you for your thoughtful review and clear feedback.

# Reviewer report: Reviewer response for version 1

**DOI:** 10.5256/f1000research.134501.r154972

**Published:** 2022-11-23

**Authors:** Mehak Pahwa

**Affiliations:** 1 Department of Psychiatry and Behavioral Sciences, University of Louisville, Louisville, KY, USA

I agree with the argument of measuring the level at 12 hrs after the last dose of both immediate release and extended-release. However, I don't understand why the correspondence is on a paper published in 2014 and there is only one citation to support it.  There are ample examples to support this view and it doesn't add anything new to the literature.

Are arguments sufficiently supported by evidence from the published literature or by new data and results?

Partly

Is the conclusion balanced and justified on the basis of the presented arguments?

Partly

Is the rationale for commenting on the previous publication clearly described?

Yes

Are any opinions stated well-argued, clear and cogent?

Partly

Reviewer Expertise:

Mood disorders, women mental health, geriatric psychiatry, bipolar disorder

I confirm that I have read this submission and believe that I have an appropriate level of expertise to confirm that it is of an acceptable scientific standard, however I have significant reservations, as outlined above.

# Response

**Published:** 2022-11-29

**Authors:** Kevin J Black

**Affiliations:** Washington University in St Louis, St. Louis, Missouri, USA

I thank Dr. Pawha for agreeing with my main point and for her thoughtful question about why this article exists. Please let me explain. The raison d'être for this article is that residents kept pulling out the referenced paper by Reddy and Reddy from PubMed to justify drawing lithium levels later for patients taking slow-release formulations. So how could I get an opposing view to future clinicians who searched PubMed to answer this question? The journal the original article appeared in does not accept late correspondence, the NLM sadly ended their PubMed Commons experiment, and few physicians use PubPeer. In short, I saw no solution other than to publish a letter like this one that PubMed would index and show as a "Comment in" link under the original abstract.
